# Transregulation of microRNA miR-21 promoter by AP-1 transcription factor in cervical cancer cells

**DOI:** 10.1186/s12935-019-0931-x

**Published:** 2019-08-15

**Authors:** Sacnite del Mar Díaz-González, Eduardo Daniel Rodríguez-Aguilar, Angélica Meneses-Acosta, Viviana Valadez-Graham, Jessica Deas, Claudia Gómez-Cerón, Carlos Alberto Tavira-Montalván, Alitzel Arizmendi-Heras, Julián Ramírez-Bello, Mario Enrique Zurita-Ortega, Berenice Illades-Aguiar, Marco Antonio Leyva-Vázquez, Gloria Fernández-Tilapa, Víctor Hugo Bermúdez-Morales, Vicente Madrid-Marina, Mauricio Rodríguez-Dorantes, Carlos Pérez-Plasencia, Oscar Peralta-Zaragoza

**Affiliations:** 1 Academic Unit of Biological Chemical Sciences, Guerrero Autonomous University, Av. Lázaro Cárdenas S/N, Col. Haciendita, 39070 Chilpancingo, Guerrero Mexico; 2 Direction of Chronic Infections and Cancer, Research Center in Infection Diseases, National Institute of Public Health, Av. Universidad No. 655, Cerrada los Pinos y Caminera. Col. Santa María Ahuacatitlán, 62100 Cuernavaca, Morelos Mexico; 3 Pharmaceutical Biotechnology Laboratory, Faculty of Pharmacy, Autonomous University of Morelos State, Av. Universidad No. 1001, Col. Chamilpa, 62010 Cuernavaca, Morelos Mexico; 4 Biotechnology Institute, National Autonomous University of México, Av. Universidad 2001, Col. Chamilpa, 62210 Cuernavaca, Morelos Mexico; 5 Endocrine and Metabolic Disease Unit Research, Hospital Juárez of México, Av. Instituto Politécnico Nacional 5160, Col. Magdalena de las Salinas, 07760 Ciudad de México, Mexico; 6 National Institute of Genomic Medicine, Periférico Sur No. 4809, Col. Arenal Tepepan, 14610 Ciudad de México, Mexico; 7 Oncogenomics Laboratory, National Cancer Institute of Mexico, Av. San Fernando No. 22, Col. Sección XVI, 14080 Ciudad de México, Mexico; 8 Biomedicine Unit, FES-Iztacala UNAM, Av. De los Barrios S/N. Col. Los Reyes Iztacala, 54090 Tlalnepantla de Baz, Estado de México Mexico

**Keywords:** AP-1, Cervical cancer, HPV, miR-21

## Abstract

**Background:**

Gene expression profiles have demonstrated that miR-21 expression is altered in almost all types of cancers and it has been classified as an oncogenic microRNA. Persistent HPV infection is the main etiologic agent in cervical cancer and induces genetic instability, including disruption of microRNA gene expression. In the present study, we analyzed the underlying mechanism of how AP-1 transcription factor can active miR-21 gene expression in cervical cancer cells.

**Methods:**

To identify that c-Fos and c-Jun regulate the expression of miR-21 we performed RT-qPCR and western blot assays. We analyzed the interaction of AP-1 with miR-21 promoter by EMSA and ChIP assays and determined the mechanism of its regulation by reporter construct plasmids. We identified the nuclear translocation of c-Fos and c-Jun by immunofluorescence microscopy assays.

**Results:**

We demonstrated that c-Fos and c-Jun proteins are expressed and regulate the expression of miR-21 in cervical cancer cells. DNA sequence analysis revealed the presence of AP-1 DNA-binding sites in the human miR-21 promoter region. EMSA analyses confirmed the interactions of the miR-21 upstream transcription factor AP-1. ChIP assays further showed the binding of c-Fos to AP-1 sequences from the miR-21 core promoter in vivo. Functional analysis of AP-1 sequences of miR-21 in reporter plasmids demonstrated that these sequences increase the miR-21 promoter activation.

**Conclusions:**

Our findings suggest a physical interaction and functional cooperation between AP-1 transcription factor in the miR-21 promoter and may explain the effect of AP-1 on miR-21 gene expression in cervical cancer cells.

## Background

In recent years, there has been great effort to elucidate the precise mechanisms involved in carcinogenesis, as well as to identify which genes are involved. Understanding of the complexity of tumor cells has been significantly enhanced with the discovery of genes producing small non-coding RNAs known as microRNAs. Moreover, it has become evident that abnormalities in microRNA expression can contribute to carcinogenesis [1]. MicroRNAs are non-coding RNAs that silence the expression of target genes at the posttranscriptional level by binding to 3′-UTRs of mRNAs and cleaving target mRNAs or repressing target mRNA translation [2, 3]. Although the effect of microRNAs on the initiation and progression of cancer has been well documented, the molecular regulatory mechanisms of microRNAs involved in cancer are poorly understood. MicroRNA biosynthesis is regulated at different levels, and transcriptional regulation at the promoter level is similar to cellular genes. Most DNA binding elements and transcription factor binding sites in microRNA promoter regions are the same as those that control protein-coding genes. However, transcription of primary microRNA transcripts can be dynamically regulated in response to stimulation by different transcription factors, and this process can be altered during carcinogenesis [4]. Thus, microRNAs and transcription factors can cause biological alterations in tumor cells leading to the event cascades of regulatory genetic networks and represent a challenge in the study of carcinogenesis.

Studies have uncovered several transcription factors as pioneer factors that can affect or even cause biological changes required in the initial steps of the transformation, invasion and metastasis cascades [5]. For instance, AP-1 is an early transcriptional regulator mainly composed of c-Fos and c-Jun family members, which bind to variations of the consensus DNA binding sequence TGAG/CTCA, usually located in promoter regions of target genes [6, 7]. An effect of the induction of both c-Fos and c-Jun oncogenes in response to upstream oncogenic signal transduction cascades is that AP-1 activity is increased in many tumor cells and transformed cell lines [8, 9]. By this molecular mechanism, AP-1 coordinates a complex program of gene expression involved in the processes of transformation, invasion and metastasis that constitute the malignancy phenotype [10]. Thus, enhanced AP-1 activity is associated with cellular oncogenic properties, and may be involved in the regulation of miR-21 transcriptional activity. The expression of microRNA miR-21 has been found to be altered in almost all types of cancers and it has been classified as an oncogenic microRNA or oncomir [11–13]. Due to the critical functions of its target proteins in various signaling pathways, miR-21 is an attractive target for genetic regulation analysis in cancer. The ability of AP-1 and miR-21 to mediate the tumorigenesis process in a variety of cell contexts, their role in oncogene biological activity, and their relevance to cell invasion during metastasis, reflect the critical function that AP-1 and miR-21 play in cancer progression.

It is well established that persistent infection with high-risk oncogenic HPV is the main etiologic agent and initial step in cervical carcinogenesis; however, other factors are required for the development and progression of the malignant phenotype [14]. An event that occurs in HPV-associated carcinogenesis is the global perturbation of cellular gene expression [15]. Although the relationship between HPV infection and cervical cancer has been well documented [16, 17], the detailed regulatory genetic networks of events leading from HPV infection to tumor development have yet to be elucidated. Despite the fact that we have a partial understanding of the molecular mechanisms responsible for microRNA gene regulation, the complete process remains to be clarified. Hence, the enhancement of AP-1 and miR-21 reflects robust regulatory genetic networks, which can contribute to oncogenic potential.

HPV16 and HPV18 are present in 70% of squamous cervical carcinomas [14]. For this reason, cell lines of cervical cancer derived from patients infected with those viral types have been intensively studied. In present study, we used human cervical cancer cells transformed with HPV16 and HPV18 as a cervical cancer model to investigate the impact of the AP-1 complex on the transcriptional activity of the miR-21 promoter. This experimental design allowed the identification of the transcriptional regulation mechanism of the miR-21 promoter in the malignancy phenotype regardless of the presence of intrinsic variations, such as the cell transformation by HPV. The principal novel contribution of our data is to propose that AP-1 transcription factor specifically upregulates miR-21 promoter activity in human cervical cancer cells, which can explain in part miR-21’s increased expression during cervical cancer development.

## Materials and methods

### Cell lines and culture conditions

Human cervical cancer cells HPV16+ (SiHa cells), HPV 18+ (HeLa cells), HPV− (C-33A cells) and human epidermal primary keratinocytes (HaCaT cells); were obtained from ATCC. The cell lines were cultured in DMEM medium (Invitrogen, Carlsbad, CA) with 10% FBS, 100 U/ml penicillin/100 μg/ml streptomycin, 2 mM l-glutamine, 250 ng/ml fungizone, and maintained at 37 °C in 5% CO_2_.

### Real-time RT-PCR analysis

SiHa and HaCaT cells stimulated with 10 ng/ml PMA (phorbol 12-myristate 13-acetate, ID SC3576A, Santa Cruz Biotechnology, Santa Cruz, CA) and 50 μM SR11302 (ID sc-204295), were harvested and processed for total RNA isolation using TriPure isolation reagent (Roche, Indianapolis, IN) according to the manual. For quantitative analysis of miR-21 expression, real-time RT-PCR analyses were performed using TaqMan pri-miRNA assays (Applied Biosystems, Foster, CA) according to the manual. Homo sapiens miR-21 gene expression [has-miR-21 NCBI: NR_029493.1] was measured using the stem-loop RT, sense and antisense primers previously reported [18]. Homo sapiens RNU44 small (ID 001094; Applied Biosystems. Foster City, CA) [has-RNU44, NCBI: NR_002750.2] was measured using the sense and antisense primers previously reported [19]. A total of 10 ng of RNA were used to reverse transcribed to cDNA. Resulting cDNA was used for quantitative real-time PCR using TaqMan microRNA assay. The expression of miR-21 was determined using microRNAs specific TaqMan probe assays (ID 000397; Applied Biosystems. Foster City, CA) in an ABI 7500 system for real-time PCR (Applied Biosystems, Foster City, CA). The reverse transcription reaction conditions were 42 °C for 30 min and 70 °C for 15 min. The PCR reaction amplification conditions were 95 °C for 10 min, 95 °C for 15 s and 60 °C for 1 min for 40 cycles. The miR-21 relative expression levels were normalized to the expression of endogenous control RNU44 and was calculated using the 2^−ΔΔCt^ method. All RT-PCR assays were performed in duplicate.

### Western blot assays

After of treatment with 10 ng/ml PMA and 50 μM SR11302, SiHa, HeLa, C-33A and HaCaT cells were harvested and proteins were isolated for western blot assays. IgG mouse monoclonal antibodies SC-166940HRP (E8) and SC-74543HRP (G4) were used to detect human c-Fos and c-Jun proteins respectively. Human beta-actin protein was detected using IgG polyclonal antibody sc-1616-HRP (Santa Cruz, Biotechnology, Santa Cruz, CA). After the peroxidase coupled secondary goat antibody mouse anti-IgG was added, bound antibodies and protein were detected by enhanced chemiluminescence using the reagent SuperSignal West Pico Chemiluminescent Substrate (Thermo Scientific, Rockford, IL, USA). The membranes were subjected to autoradiography with an intensifier screen.

### DNA–protein interaction analysis by EMSA assays

EMSA assays were performed to identify the DNA–protein interactions. Briefly, probes were oligonucleotides duplex containing the AP1D, AP1M and AP1P binding sites from miR-21 promoter region, which were 5′-end labeled with biotin (Integrated DNA Technologies, Coralville, IO). The sequences for AP1D were sense 5′-TGT-TAA-TCA-CTG-ACT-TCT-GAC-TAG-TGG-3′ and antisense 5′-CCA-CTA-GTC-AGA-AGT-CAG-TGA-TTA-ACA-3′. The sequences for AP1M were sense 5′-TGG-ATA-AGG-ATG-ACG-CAC-AGA-TTG-TCC-3′ and antisense 5′-GGA-CAA-TCT-GTG-CGT-CAT-CCT-TAT-CCA-3′. The sequences for AP1P were sense 5′-TTA-CTA-GGG-ATG-ACA-CAA-GCA-TAA-GTC-ATT-T-3′ and antisense 5′-AAA-TGA-CTT-ATG-CTT-GTG-TCA-TCC-CTA-GTT-A-3′. Nuclear extracts of SiHa, HeLa, C-33A and HaCaT cells were obtained according to NE-PER kit protocol (Pierce, Rockford, IL). Binding reactions were performed at 4 °C for 30 min using chemiluminescent nucleic acid detection module protocol. (Thermo Scientific, Waltham MA). For competition assays, 1000-fold molar excess of AP-1 autologous unlabeled probes and equimolar NF-kB heterologous competitor unlabeled probes were added 30 min before incorporating the AP1D, AP1M and AP1P labeled probes. For super-band shift assays, DNA–protein complexes were allowed to form prior to the addition of 5 μg of polyclonal anti-c-Fos antibody (sc-52, Santa Cruz Biotechnology, Santa Cruz, CA). DNA–protein complexes were resolved in low-isotonic strength on non-denaturing 7% PAGE containing 0.5× TBE for 3 h at 200 volts and transferred to PVDF membrane in trans-blot semi-dry chamber (Bio-Rad, Hercules CA). Membrane was exposed to UV light for 10 min and subjected to autoradiography. DNA–protein complexes were detected by using light chemiluminescent EMSA kit (Pierce, Rockford, IL).

### In vivo DNA–protein interaction analysis by ChIP-PCR assays

ChIP-PCR assays were carried out. Briefly, 1 × 10^7^ cells were sonicated in a 30 on/30 off program and the chromatin solution was precleared with the addition pre-cleaning with magnetic beads for 6 h at 4 °C to obtain chromatin fragments ranging from 100 bp-500 bp. The anti-c-Fos antibody was added. Mock was used an IgG (anti-goat IgG) as an irrelevant antibody. Magnetic beads were added and incubated for 2 h at 4 °C. After RNase I and proteinase K treatments and reversal of cross-linking, DNA isolation was carried out with phenol–chloroform-isoamyl alcohol. End-point PCR amplifications were carried out with oligos to AP1D, AP1M and AP1P sequences of miR-21 promoter. MMP1 collagenase gene was amplified as positive control using sense 5′-CGG-GGT-ACC-CAT-CTT-GTT-TGA-AGT-3′ and antisense 5′-CCC-AAG-CTT-CTT-GCT-GCT-CCA-ATA-C-3′ primers which amplified a product of 210 bp. Actin housekeeping gene was used as internal control.

### Cloning strategy of miR-21 promoter and reporter plasmid constructs

Homo sapiens hsa-miR-21 gene promoter [Ensembl: ENSG00000284190.1] was obtained by end-point PCR amplification of genomic DNA from HaCaT cells using TriPure isolation reagent (Roche, Indianapolis, IN). PCR reaction amplification conditions were 95 °C for 10 min, 95 °C for 1 min, 55 °C for 30 s and 72 °C for 1 min for 35 cycles followed by 72 °C for 10 min. The promoter region that contains deleted the AP-1 sequences, was obtained by gBlock synthesis (Integrated DNA Technologies, Coralville, IO) and a 433 bp DNA fragment was obtained and was cloned to generate the pG0AP1MIR21 plasmid. The promoter region that contains AP1P sequence was obtained using sense 5′-CGG-GGT-ACC-GAG-AAG-AGG-GGA-CAA-GTC-3′ and antisense 5′-GCC-AAG-CTT-CAA-AAT-CTC-TCC-CAC-CAA-C-3′ primers and a 284 bp DNA fragment was obtained and was cloned to generate the pG1AP1MIR21 plasmid. The promoter region that contains the AP1M and AP1P sequences was obtained using sense 5′-CGG-GGT-ACC-CTC-CCA-AGT-TTG-CTA-ATG-C-3′ and antisense 5′-GCC-AAG-CTT-CAA-AAT-CTC-TCC-CAC-CAA-C-3′ primers and a 374 bp DNA fragment was obtained and was cloned to generate the pG2AP1MIR21 plasmid. The promoter region that contains the AP1D, AP1M and AP1P sequences, was obtained using sense 5′-CGG-GGT-ACC-CTG-TGC-AAA-CTG-TCT-ACC-3´ and antisense 5′-GCC-AAG-CTT-ACA-TGT-CTG-GGA-GAA-ACC-3′ primers and a 456 bp DNA fragment was obtained and was cloned to generate the pG3AP1MIR21 plasmid. Each miR-21 promoter region was cloned in *Kpn* I y *Hind* III restriction sites of pGL2-Basic reporter vector (Promega, Madison, WI). Plasmids were isolated with PureYield plasmid midiprep system (Promega, Madison, WI) and the integrity was verified by DNA sequencing in Genetic Analyzer 3500xl equipment (Applied Biosystems, Foster, CA).

### Cellular transfection and luciferase activity assays

SiHa, HeLa, C-33A and HaCaT cells were transiently transfected with pG0AP1MIR21, pG1AP1MIR21, pG2AP1MIR21, and pG3AP1MIR21 reporter plasmids. Briefly, 1 day before of transfection assay, the cells were plated at 1 × 10^5^ cells per well in a six-well plate containing 2 ml of DMEM with 10% FBS. At the time of transfection, the plasmids and Fugene reagent (Promega, Madison, WI) were diluted in DMEM and incubated for 20 min. Cells were incubated with 5 μg of plasmids and 10 μl of Fugene for 4 h, rinsed and replenished with DMEM containing 10% FBS. After 48 h of transfection, cells were harvested and lysed with 100 µl cold lysis buffer. Cellular extracts were collected and 50 µg total proteins were used to determine luciferase activity using the Dual-Glo luciferase assay system (Promega, Madison WI) in Glomax Multidetection equipment. Luminescence was calculated to normalize results with respect to transfection efficiency of pGL2-Basic and pGL2-Control plasmids.

### Immunofluorescence microscopy assays

Immunofluorescence microscopy arrays were carried out to determine localization of AP-1 protein. Briefly, 1 day before the assay, the cells were plated at 1 × 10^5^ cells per well in chamber slide containing 2 ml of DMEM with 10% FBS. Cells were fixed with 4% paraformaldehyde and permeabilized with 0.3% saponin. To investigate the cellular localization of AP-1, anti-c-Fos (sc-74543-FITC) and anti-c-Jun-specific antibodies FITC-conjugated (sc-166940-FITC, Santa Cruz Biotechnology, Santa Cruz, CA) were used. For nuclear staining, 0.5 µg/ml Hoechst solution was added. Slides were mounted in Citifluor. A Nikon Elipse 400 epifluorescence microscope was used to analyze the samples by FITC using the 40× Fluor objectives.

### Statistical analysis

All experiments were performed at least three times. The data were analyzed and one-way ANOVA and post-test Tukey was carried out to compare frequencies between the different experimental groups. *P*-values less than 0.05 were considered to be statistically significant and were indicated with an asterisk (*).

## Results

### Constitutive activation of AP-1 induces miR-21 expression in human cervical cancer cells

**Fig. 1:**
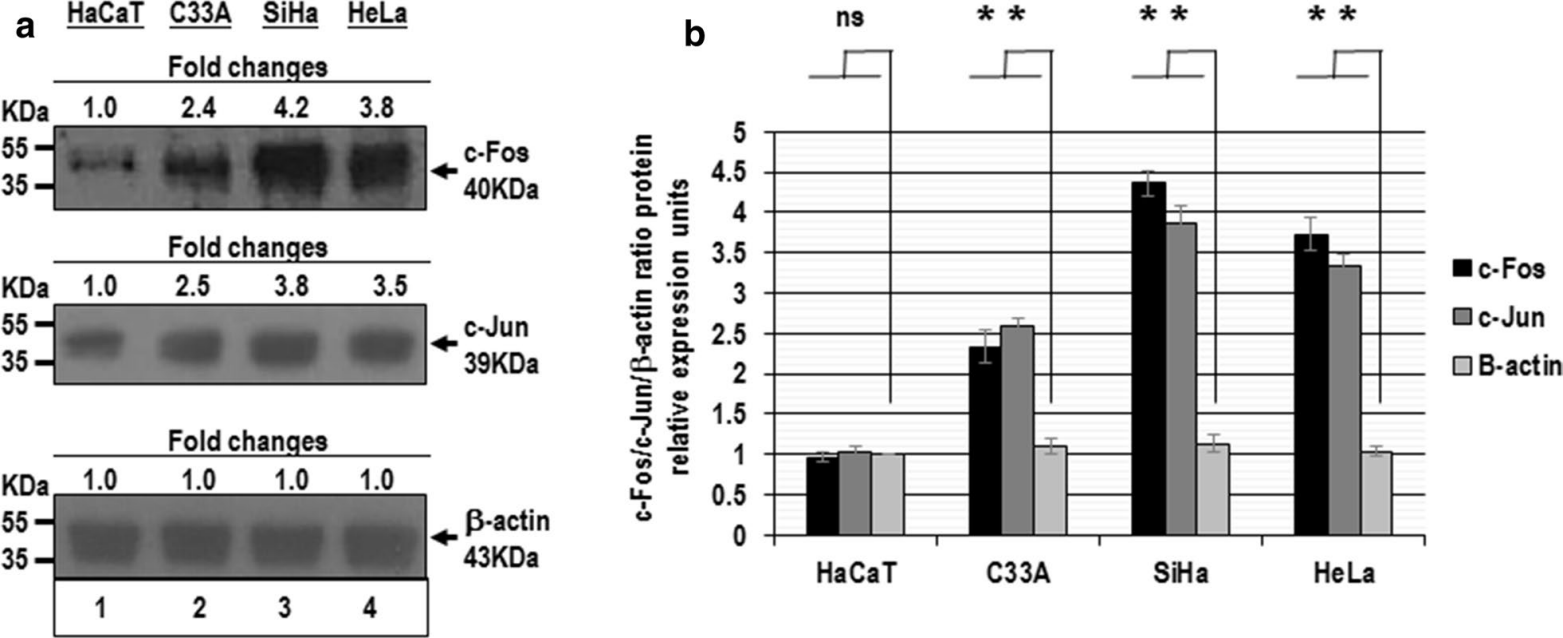
Analysis of c-Fos and c-Jun protein expression in cervical cancer cells. Total cellular proteins were obtained from 1 × 10^5^ HaCaT (lane 1), C-33A (lane 2), SiHa (lane 3) and HeLa cells (lane 4) per well in a six-well plate containing DMEM at 37 °C with 10% FBS in 5% CO_2_. The proteins were separated in 12% SDS-PAGE and were transferred to nitrocellulose membranes, which were incubated with each antibody. **a** The amount of similar proteins analyzed in the immunoblots. The anti-beta-actin antibody was included as control. **b** The immunoblot bands digitalized and analyzed by densitometer. The data were analyzed by c-Fos/c-Jun/beta-actin fold change in relative expression units (mean ± SE), not statistically significant (ns) and P values < 0.05 are indicated with asterisks. The data are representative of three independent experiments

In order to analyze the c-Fos and c-Jun expression in different cervical cancer cells, we measured the level basal of c-Fos and c-Jun protein expression in C-33A, SiHa, HeLa cells, corresponding to HPV-, HPV16 and HPV18 infection respectively, and HaCaT non-tumoral cervical cancer cells were used as control. As shown in Fig. 1a we found a higher expression of these proteins in C-33A, SiHa, HeLa cell lines, two, four and three-fold respectively. While, in HaCaT cells we only observed a slight level of c-Fos and c-Jun protein expression. We observed statistically significant differences in the c-Fos and c-Jun protein expression in C-33A, SiHa, HeLa cells with respect to the beta-actin protein expression in HaCaT cells.

**Fig. 2:**
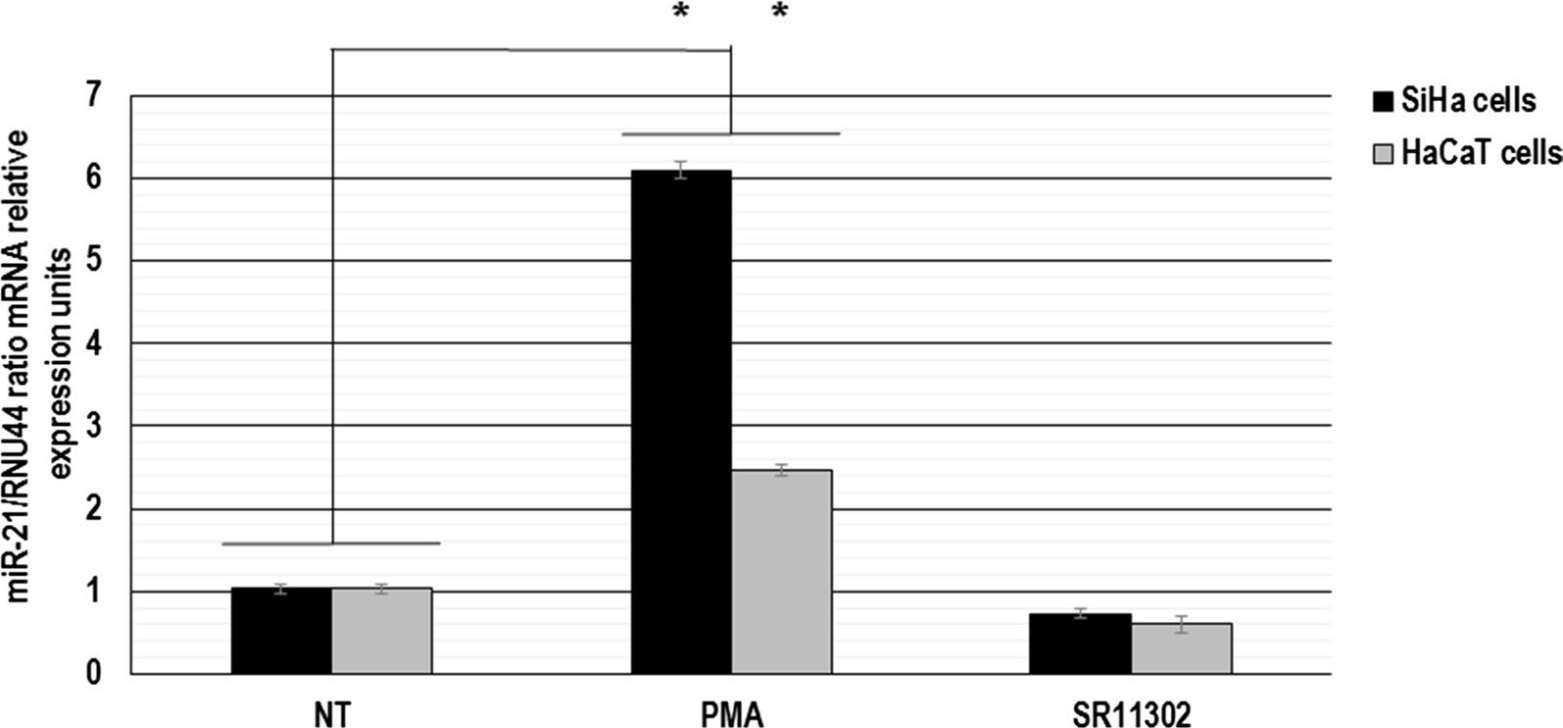
MiR-21 gene expression analysis after constitutive activation of AP-1. Total RNA and cDNA synthesis were obtained from 1 × 10^5^ SiHa and HaCaT cells per well in a six-well plate containing DMEM at 37 °C with 10% FBS in 5% CO_2_ after 60 min of non-treatment (NT) or treatment with 10 ng/ml PMA and 50 μM SR11302. The miR-21 gene expression was analyzed by real-time qRT-PCR and relative expression of miR-21 was calculated using the 2^−∆∆Ct^ method. The RNU44 gene expression was used as control. The data were analyzed by mRNA miR-21/mRNA RNU44 ratio in relative expression units. The values are presented as mean ± SD and P values < 0.05 are indicated with asterisks. The data are representative of three independent experiments

It has been well established that activation of AP-1 transcription factor is induced by cell stimulation with PMA, while SR11302 is an inhibitor of AP-1. In order to clarify the status of miR-21 gene expression under AP-1 regulation in cervical tumor cells, we evaluated the expression level of miR-21 in SiHa and HaCaT cells treated with PMA and SR11302 by real-time qRT-PCR and we found a heterogeneous expression of this microRNA. As shown in Fig. 2, we observed a differential expression of miR-21 gene in tumor cells when were treated with PMA. The SiHa cells showed an increase of miR-21 gene expression of six-fold compared with non-treated cells, which was statistically significant. To evaluate whether miR-21 expression is also induced in normal cells by treatment with PMA, we carried out real-time qRT-PCR assays in HaCaT cells. We observed an increase of two-fold of miR-21 gene expression in HaCaT cells compared with non-treated cells, which was statistically significant (Fig. 2). When we analyzed miR-21 expression levels in SiHa and HaCaT cells treated with SR11302, we identified inhibition of miR-21 gene expression in both cell types, but we did not observe a statistically significant decrease compared with non-treated cells. The control RNU44 RNA expression level did not show significant changes under these same conditions.

**Fig. 3:**
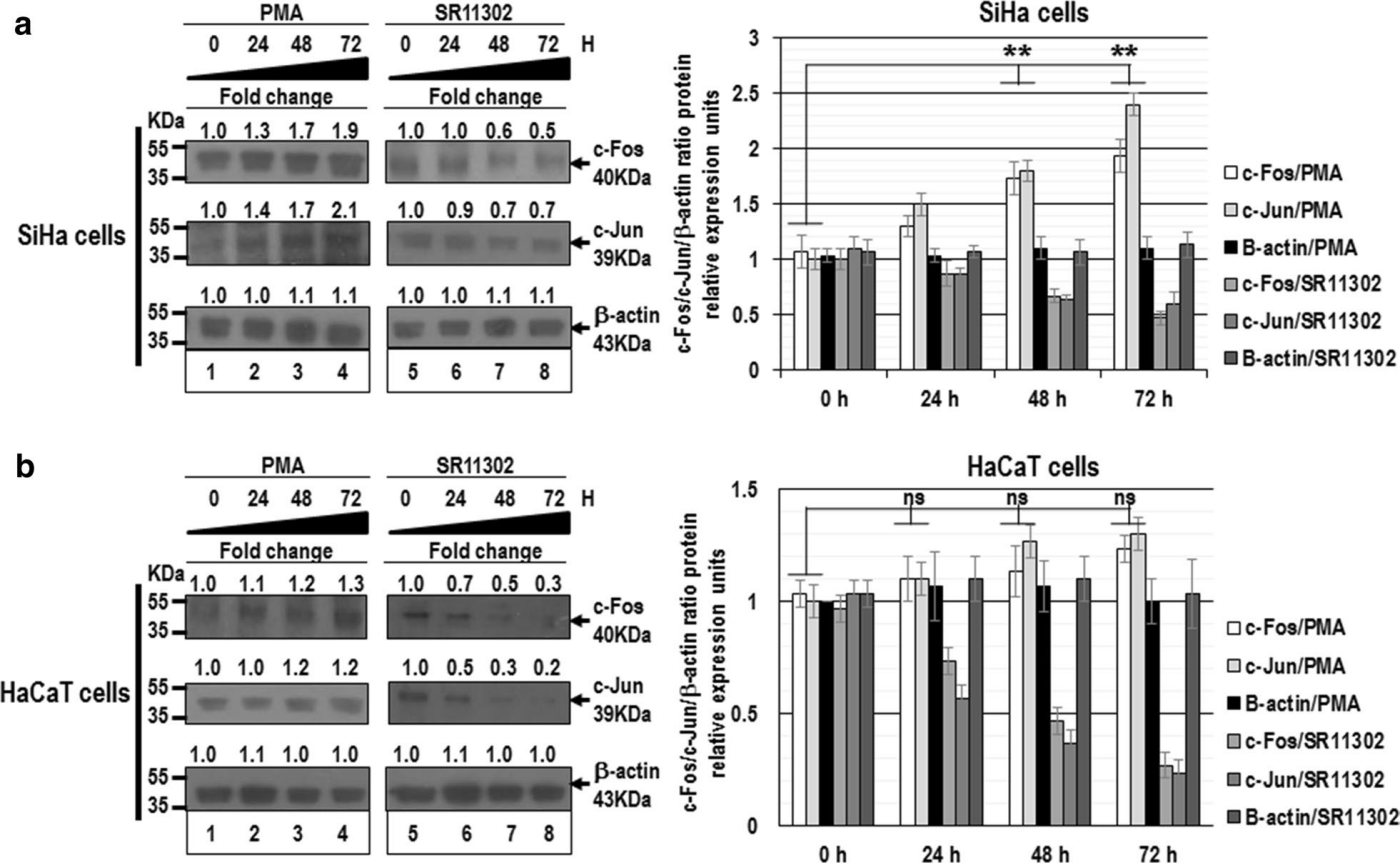
Analysis of c-Fos and c-Jun protein expression after constitutive activation of AP-1. Total cellular proteins were obtained from 1 × 10^5^ SiHa (**a**) and HaCaT cells (**b**) per well in a six-well plate containing DMEM at 37 °C with 10% FBS in 5% CO_2_ after 0, 24, 48 and 72 h treated with 10 ng/ml PMA (lanes 1–4) or 50 μM SR11302 (lanes 5–8). The proteins were separated in 12% SDS-PAGE and were transferred to nitrocellulose membranes, which were incubated with each antibody. Similar amount of proteins were analyzed in the immunoblots. The anti-beta-actin antibody was included as control. The immunoblot bands were digitalized and analyzed by densitometer. The data were analyzed by c-Fos/c-Jun/beta-actin fold change in relative expression units (mean ± SE), not statistically significant (ns) and P values < 0.05 are indicated with asterisks. The data are representative of three independent experiments

To confirm that miR-21 gene expression pattern was consistent with constitutive activation of AP-1, we evaluated the c-Fos and c-Jun protein expression by western blot assays. When SiHa cells were treated with PMA, the c-Fos and c-Jun expression level increased approximately two-fold compared with cells untreated with PMA, which was statistically significant (Fig. 3a). We did not observe significant changes in c-Fos and c-Jun protein expression in HaCaT cells treated with PMA (Fig. 3b). We used beta-actin protein as control, and we did not observe significant changes in expression protein levels, as well.

These data demonstrate that c-Fos and c-Jun proteins are expressed of differential manner in cervical cancer cells compared with normal keratinocytes and suggest that the AP-1 complex can exist as c-Fos and c-Jun heterodimers in human cervical cancer cells. Furthermore, our results indicate that constitutive activation of AP-1 transcription factor can induce the expression of miR-21 in human cervical cancer cells transformed with oncogenic HPVs.

### c-Jun and c-Fos are translocated into the nuclei of cervical cancer cells

**Fig. 4:**
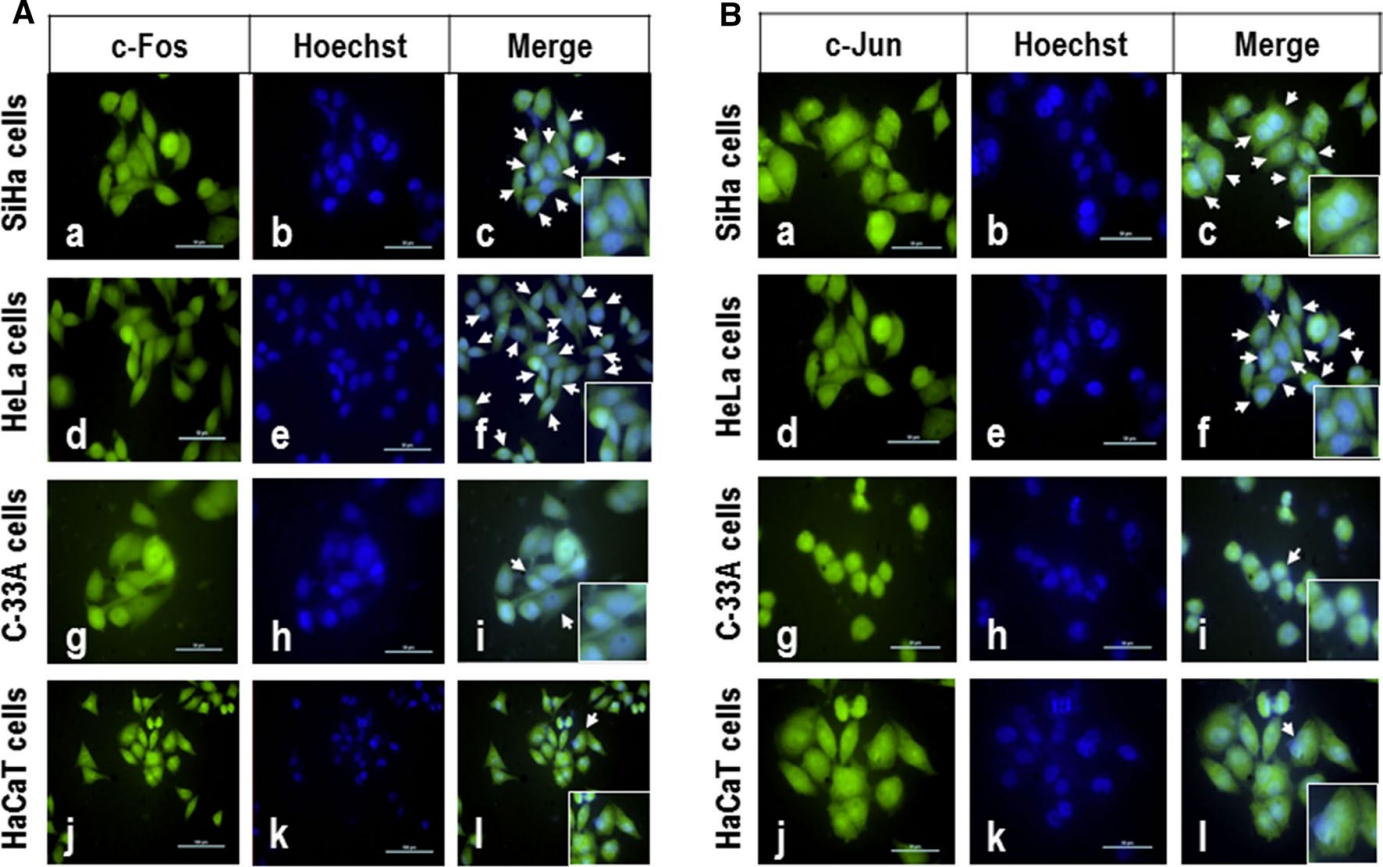
Nuclear translocation of c-Fos and c-Jun proteins in cervical cancer cells. SiHa, HeLa, C-33A and HaCaT cells were fixed with paraformaldehyde, permeabilized with saponin, stained with anti-c-Fos or anti-c-Jun antibodies FITC-conjugated, and were analyzed by immunofluorescence microscopy by FITC in a Nikon Elipse 400 epifluorescence microscope using the 40× Fluor objective. The arrows indicate the nuclear localization of c-Fos (**A**) and c-Jun protein (**B**) in SiHa (**a**–**c**), HeLa (**d**–**f**), C-33A (**g**–**i**) and HaCaT cells (**j**–**l**). Scale bar, 50 μm. The data are representative of three independent experiments

The above results confirmed that c-Fos and c-Jun proteins are expressed in cervical cancer cells and that they could effectively trans-regulate the miR-21 gene promoter. Therefore, we sought to determine whether c-Fos and c-Jun proteins form heterodimers and whether those heterodimers translocate into the nuclei of cervical cancer cells. For this purpose, we directly analyzed the intracellular behavior of c-Fos and c-Jun proteins using immunofluorescence microscopy assays. Results revealed that 60% of SiHa cells had positive nuclear staining for c-Fos protein (Fig. 4A, a–c), that 70% of HeLa cells had positive nuclear staining (Fig. 4A, d–f), and that 30% of C-33A cell had positive nuclear staining (Fig. 4A, g–i). Interestingly, we observed a marginal expression of c-Fos nuclear translocation in HaCaT cells with fewer than 10% with positive nuclear staining for c-Fos protein (Fig. 4A, j–l). Similar results were obtained for c-Jun protein in the same cell types (Fig. 4B). These observations demonstrated the presence of c-Fos and c-Jun in the cell nucleus, suggesting that nuclear translocation occurs and may trans-regulate the miR-21 gene promoter in human cervical cancer cells.

### AP-1 transcription factor binds to the miR-21 promoter region

**Fig. 5:**
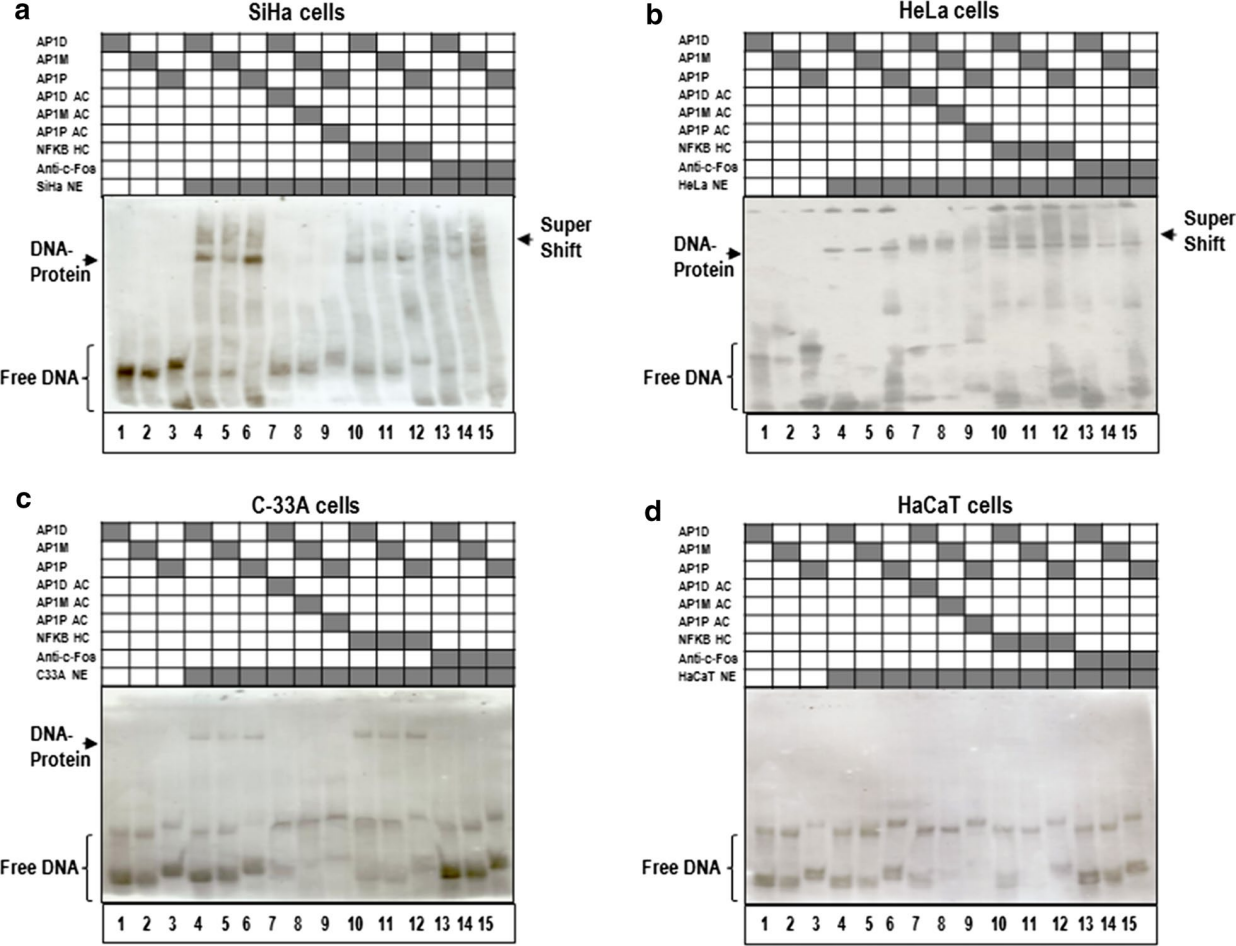
Binding analysis of AP-1 recognition sequences from the human miR-21 promoter in cervical cancer cells. The AP1D, AP1M and AP1P probes were 5′-ends labeled with biotin and were incubated in absence (lanes 1–3) or in presence of nuclear extracts (NE; lanes 4–15), from SiHa (**a**), HeLa (**b**), C-33A (**c**) and HaCaT cells (**d**). The nuclear extracts were pre-incubated with 1000-fold molar excess of unlabeled AP1 probes as specific autologous competitor (AC; lanes 7–9) or with equimolar concentration of NF-kB probe as heterologous competitor (HC; lanes 10–12), before to add the labeled AP1 probes. In super-band shift assays, DNA–protein complexes were allowed to form prior to the addition of anti-c-Fos antibody (Anti-c-Fos; lanes 13–15). The arrows indicate the formation of specific retarded DNA–protein and super-band shift retarded complexes in each case, and free DNA is indicated. The data are representative of three independent experiments

To comprehensively describe the function of AP-1 transcription factor in miR-21 gene expression in human cervical cancer cells, we performed bioinformatics analysis based in the TFSEARCH website. This analysis revealed the presence of several conserved enhancer recognition elements in the 5′-end regulatory region of the miR-21 gene, including binding sites for AP-1, which herein are denominated as AP-1 Distal (AP1D), AP-1 Medial (AP-1M) and AP-1 Proximal (AP1P). To examine the binding of AP-1 transcription factor in the regulation of the miR-21 promoter, we performed EMSA assays with AP1D, AP1M and AP1P probes using nuclear extracts from SiHa (Fig. 5a), HeLa (Fig. 5b), C-33A (Fig. 5c) and HaCaT cells (Fig. 5d). We identified several retarded DNA–protein complexes in SiHa, HeLa and C-33A cells, indicating that AP-1 binding sites from miR-21 gene induced the formation of DNA–protein migrating complexes with a similar mobility to that generated by the AP-1 consensus sequence (Fig. 5a–c; lanes 4 to 6). To verify the specificity of nuclear proteins binding to the miR-21 promoter, we carried out autologous competition assays with 1000-fold molar excess of unlabeled AP1D, AP1M and AP1P probes before adding the same labeled probes (Fig. 5a–c; lanes 7 to 9). We observed a specific competition by AP-1 recognition sites in SiHa, HeLa and C-33A cells. Furthermore, the specificity of retarded DNA–protein complexes was examined by means of unlabeled probe containing DNA-binding sequences for NF-kB transcription factor as non-specific competitor (Fig. 5a–c; lanes 10 to 12). We did not observe competition by formation of retarded DNA–protein complexes with NF-kB probes. To confirm that members of the AP-1 transcription factor family are involved in the formation of DNA–protein complexes; we evaluated the presence of c-Fos protein in retarded DNA–protein complexes using an anti-c-Fos antibody (Fig. 5a–c; lanes 13 to 15). We found that when retarded DNA–protein complexes were pre-incubated with the anti-c-Fos antibody a faint formation of super-retarded DNA–protein complexes was observed in SiHa and HeLa cells. These super-retarded complexes were not identified in C-33A cells. To determine whether AP-1 transcription factor also recognizes the AP1D, AP1M and AP1P sequences in normal cells, we performed EMSA assays in HaCaT cells. Interestingly, we did not find formation of these retarded DNA–protein complexes in normal cells (Fig. 5d). Taken together, these data support the fact that AP1D, AP1M and AP1P sequences from the miR-21 promoter region have the ability to recruit the AP-1 transcription factor in cervical cancer cells.

### c-Fos has the ability to bind to AP-1 recognition sequences from the miR-21 promoter region in vivo

**Fig. 6:**
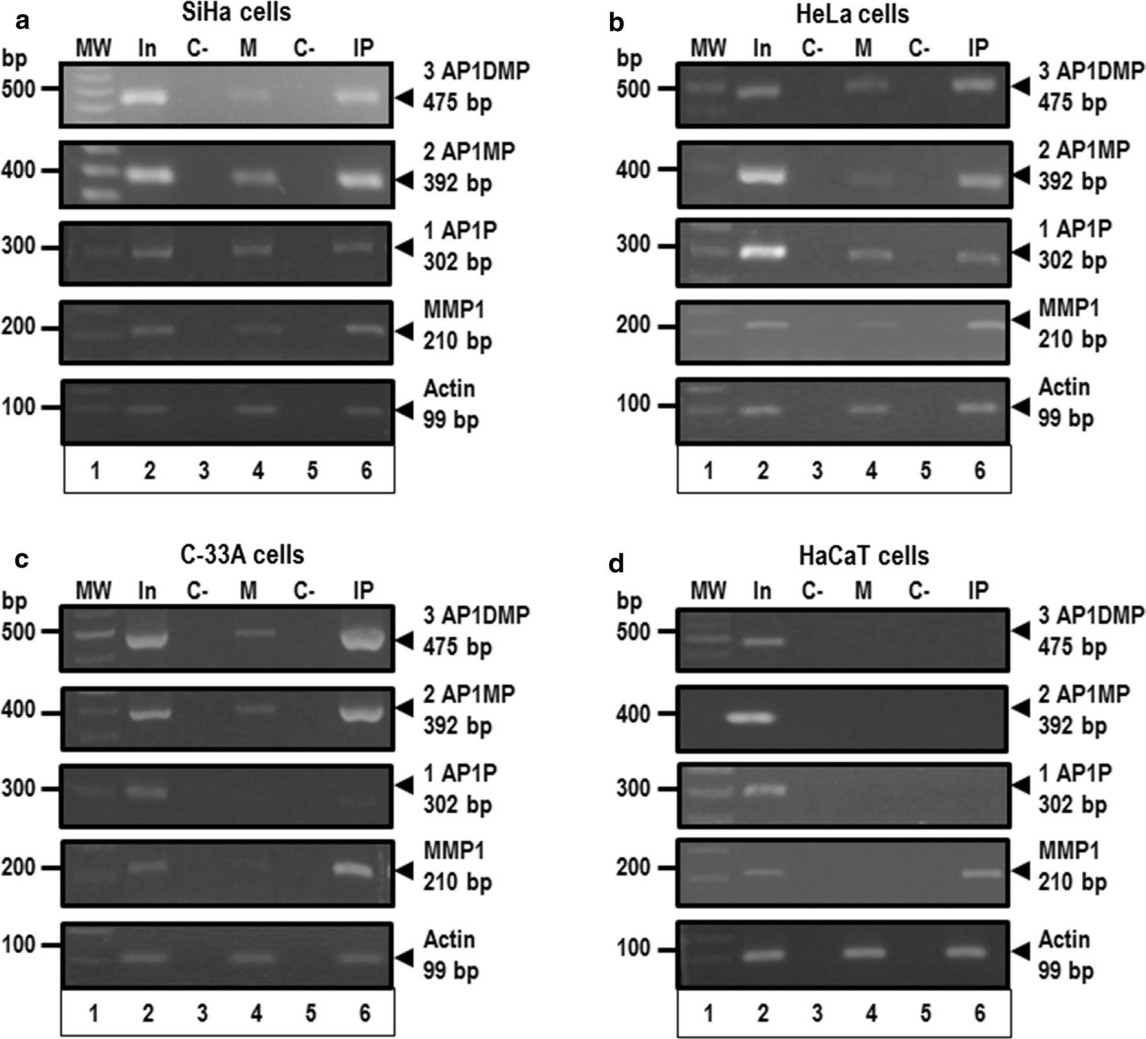
In vivo interaction analysis of AP-1 recognition sequences from the human miR-21 promoter. DNA–protein complexes from SiHa, HeLa, C-33A and HaCaT cells were established using formaldehyde and DNA was fragmented by sonication. DNA was recovered and immunoprecipitations were carried out using anti-c-Fos antibody. PCR amplification products of 475 bp DNA fragment that contains the AP1D, AP1M and AP1P sequences (3 AP1DMP), of 392 bp DNA fragment that contains AP1M and AP1P sequences (2 AP1MP), and of 302 bp DNA fragment that contains the AP1P sequence (1 AP1P) from miR-21 promoter respectively, were resolved in 1% agarose gel electrophoresis using DNA from ChIP assay to SiHa (**a**), HeLa (**b**), C-33A (**c**) and HaCaT cells (**d**). The DNA 100 bp ladder (MW; lane 1), input condition (In; lane 2), negative control of PCR reaction (C-, lanes 3 and 5), mock condition (M; lane 4), and immunoprecipitation conditions with anti-c-fos antibody (IP; lane 6) were included. The AP-1 sequence from metalloproteinase 1 gene (MMP1) as well as actin housekeeping gene were used as controls respectively. The data are representative at least three independent experiments

Tumor cells have the ability to self-renew and differentiate into downstream lineages, which depend on specialized chromatin remodeling that establishes and maintains state-specific patterns of gene expression. In order to characterize the regulatory mechanism by which the AP-1 transcription factor complex modulates miR-21 expression, we assessed whether c-Fos protein is associated in vivo with the AP-1 recognition sequences of the miR-21 promoter previously identified in cervical cancer cells. ChIP assays using an anti-c-Fos antibody was carried out in the different cervical tumor cells (Fig. 6). We found that the c-Fos protein was enriched in the promoter region that contains the three AP1D, AP1M and AP1P sequences, in SiHa (Fig. 6a, lane 6), and HeLa cells (Fig. 6b, lane 6). Similar results were obtained when we analyzed a region of miR-21 promoter that contains only two AP1M and AP1P sequences, or only the AP1P sequence. Interestingly, when we assayed a DNA fragment that contains the AP1P sequence, we did not observe the enrichment of c-Fos protein in C-33A cells (Fig. 6c, lane 6). In contrast, c-Fos protein was not enriched in the same miR-21 promoter regions in HaCaT cells, probability because HaCaT cells have an epithelial origin and have a controlled replicative potential, which allows us to distinguish between the biological events of transformation and immortalization in cancer cells (Fig. 6d, lane 6). The AP-1 sequence from metalloproteinase 1 gene (MMP1), as well as actin housekeeping gene were used as positive controls. These data show that AP-1 transcription factor, and specifically c-Fos protein, is differentially bound in vivo to AP-1 recognition sequences from the miR-21 promoter region in cervical cancer cells transformed with oncogenic HPVs. Thus, our results are consistent and support the previous findings of the EMSA experiments to explain the molecular mechanism of miR-21 gene regulation in cervical cancer cells transformed with high-risk oncogenic HPVs.

### The miR-21 promotor is trans-regulated by AP-1 transcription factor

**Fig. 7:**
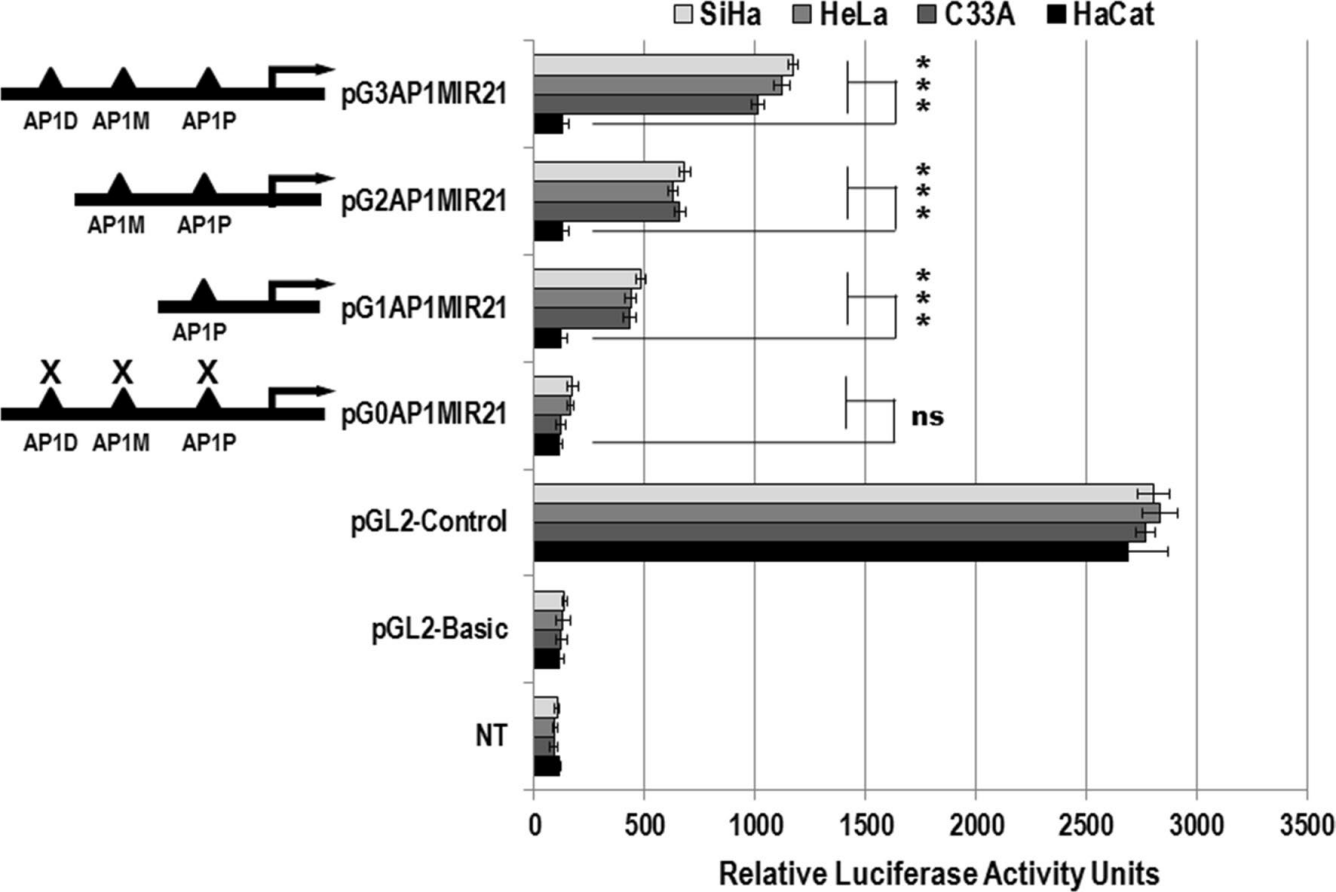
Functional analysis of miR-21 promoter activity trans-regulation by AP-1 transcription factor. SiHa, HeLa, C-33A and HaCaT cells were transiently transfected in an independent manner with pG0AP1MIR21, pG1AP1MIR21, pG2AP1MIR21 and pG3AP1MIR21 reporter plasmids that contain progressive deletions of AP1D, AP1M and AP1P sequences of miR-21 promoter. After 48 h of transfection, the luciferase activity levels were analyzed in relative luciferase activity units (mean ± SE), not statistically significant (ns) and P values < 0.05 are indicated with asterisks. The pBL2-Basic and pGL2-Control correspond to negative and positive transfection controls, respectively. NT corresponds to non-transfected cells. All transfections were repeated at least three times independently

To characterize the transcriptional regulation mechanism of miR-21 gene by AP-1 transcription factor, we analyzed miR-21 promoter activity using luciferase reporter gene containing the different AP-1 recognition sites. As shown in Fig. 7, the SiHa, HeLa, C-33A and HaCaT cells were transiently transfected with reporter plasmids that have progressive deletions of AP-1 binding sites. The results showed that the miR-21 promoter containing the three AP1D, AP1M and AP1P sequences induced the highest luciferase reporter activity, reaching the highest trans-activation levels. When the AP1D sequence was eliminated, the luciferase activity diminished up to 25%. When both AP1D and AP1M were deleted the luciferase activity decreased up to 75%, which were statistically significant with respect to the HaCaT cells. We did not observe luciferase reporter activity in the absence of all three AP1D, AP1M and AP1P sequences. Interestingly, when HaCaT cell were transfected with the same reporter plasmids, we did not observed changes in luciferase activity. These results suggest that AP1D, AP1M and AP1P sequences are transcriptionally functional in the regulatory activity of miR-21 gene promoter in human cervical cancer cells transformed by high-risk oncogenic HPVs.

## Discussion

Previously, we reported that miR-21 down-regulates the expression of target cellular genes in cervical intraepithelial neoplasia-derived cell lines [20]. In order to understanding the upstream regulatory genetic network of the miR-21 gene, we characterized the mechanism of miR-21 gene regulation in cervical cancer cells and described how this molecular pathway contributes to cervical carcinogenesis. In the present study, we demonstrated that members of the AP-1 transcription factor family, including c-Fos and c-Jun, have an essential role in miR-21 gene regulation in cervical cancer cells transformed with high-risk oncogenic HPV.

Several pieces of evidence support the idea that changes in AP-1 complex composition are involved in gene transcriptional regulation during cervical carcinogenesis. We demonstrated than miR-21 expression is induced by constitutive activation of AP-1 in cervical cancer cells (Figs. 1 and 2). AP-1 is a transcription factor involved in a wide variety of pathophysiological responses including cancer. MAPKs including ERKs, JNKs and p38 kinases are known to be the most general common signaling pathways modulating AP-1 activity in response to external stimuli. Therefore, the agents that stimulate or inhibit AP-1 activation such as PMA or SR11302, respectively, may act in the MAPKs signal pathways and in this manner regulate miR-21 expression. Interestingly, this regulatory mechanism seems to be specific to cervical cancer cell lines, since the miR-21 expression in non-tumor cell line (HaCaT cells) was not robustly affected by AP-1 transcriptional factors.

Furthermore, we found endogenous differences in each cell type, with levels of the c-Fos/c-Jun heterodimer increased in SiHa and HeLa cells (Fig. 3). These data suggest a heterogeneous behavior of AP-1 transcription factor specific to each cervical cancer cell line. Previously, De Wilde et al., reported that mRNA expression levels of c-Fos, Fra-1, Fra-2, c-Jun and JunB change at different stages during the malignant transformation of primary keratinocytes by high-risk HPV [21]. While the onset of deregulated expression varied among the AP-1 members, a shift in AP-1 complex composition toward c-Fos and c-Jun expression was favored in tumorigenic cells. They concluded that HPV-mediated transformation cell is associated with altered expression of AP-1 transcription factor among the different genes during the transition from early to late immortalization. It has been also reported that in cells that do not follow a neoplastic process, the AP-1 complex is composed mainly of c-Jun/Fra-1 heterodimers and c-Fos protein is almost absent. On the other hand, in malignant cell lines such as HeLa, SW756 and SiHa, the levels of c-Fos protein are increased and the levels of Fra-1 are decreased, resulting in a prevalent c-Jun/c-Fos dimerization pattern [22]. Our findings of increased expression of c-Fos and c-Jun in SiHa and HeLa cells provide insight into the increased expression of the c-Fos/c-Jun heterodimer in the AP-1 complex in immortal and tumorigenic cells, which closely resemble to malignant cervical lesions. Furthermore, our data suggest that constitutive activation of AP-1 results in high levels of c-Jun that could also activate the c-Fos gene promoter and induce formation of the oncogenic dimer c-Fos/c-Jun, which may be involved in the transcriptional regulation of the miR-21 promoter. In addition, we found that the c-Fos and c-Jun proteins co-localize into the nucleus of cervical cancer cells (Fig. 4). To strengthen our evidence that c-Fos and c-Jun proteins form heterodimers, and translocate into nuclei of cervical cancer cells, is necessary to carry out additional assays such as western blot with a nuclei-cytoplasm protein fractionation, or confocal microscopy assays. However, we do not have the necessary resource to perform this type of assays and this represents a limitation of our study. This finding confirms that the c-Fos and c-Jun proteins has ability to translocate into the nuclei in cervical cancer cells, and that miR-21 promoter activity can be dependent of AP-1 binding sites.

In order to enhance understanding of the AP-1/miR-21 regulatory genetic network, we analyzed the direct binding of AP-1 transcription factor to the miR-21 promoter region in human cervical cancer cells (Fig. 5). The data presented herein demonstrated that c-Fos protein is required for binding to the miR-21 promoter. Interestingly, we observed some DNA–protein signal in the autologous competition in HeLa cells. This DNA–protein signal can be explained by the different alignment of AP1D, AP1M and AP1P sequences, which can recruit different members of AP-1 transcription factor family, and/or other co-factors associated to the AP-1 complex. We found that the sequences identified as AP1D (GTTAATCAC from − 270 to − 262 nt), AP1M (GATGACG from 202 to − 196 nt) and AP1P (GATGACA from 95 to − 89 nt) possess divergent sequence motifs in comparison to the canonical AP-1 binding site (TGAG/CTCA). The AP-1 complex was originally identified as a dimeric transcription factor composed of either a homodimer or heterodimer of AP-1 family members that bind to variations of the canonical DNA binding sites TGAG/CTCA in the 5′-end promoter region of target genes [23]. Binding of AP-1 produces transcriptional synergy of DNA binding that could result in highly specific protein–protein interactions between AP-1 and other promoter-bound transcription complexes. Once the c-Fos/c-Jun heterodimer binds promoter regions, these non-consensus AP-1 sites synergistically enhance transcription, and this transcriptional machine is essential to regulation of several human genes involved in cellular homeostasis, as has been reported for other AP-1-driven promoter interactions [24]. For instance, using recombinant full-length human AP-1 dimers formed between c-Fos and c-Jun for DNA binding and transcriptional analysis, each AP-1 complex exhibits differential activity for distinct non-consensus AP-1 recognition sequences present in the long control region of HPV [25]. Our results are consistent with other research groups, with the novel finding that AP-1 binding sites in the miR-21 promoter can be selectively recognized by constitutive activation of AP-1 complex. In addition, our findings provide insight into the importance of AP-1 complex composition, the relevance of non-canonical binding sites in the miR-21 promoter region recognized by AP-1 family members, and AP-1’s impact in the malignant phenotype of cervical cancer cell lines.

Using in vivo DNA–protein interaction analysis, we demonstrated that AP-1 transcription factor is recruited to AP1D, AP1M, and AP1P sequences in a differential manner to induce the miR-21 promoter in cervical cancer cells (Fig. 6). Our data show that there are not bound levels of AP-1 in the miR-21 promoter region in HaCaT cells, while in SiHa cells, the promoter region of miR-21 is already bound by AP-1. The transcript levels of miR-21 in SiHa and HaCaT cells is similar in both cell lines without PMA treatment (Fig. 2). However, upon PMA treatment, there is a more than six-fold increase in miR-21 transcript levels in SiHa cells (Fig. 6), whereas in PMA treated HaCaT cells there is a roughly two-fold increase in miR-21. These results suggest that recruitment of AP-1 at the 3AP1DMP site is a requisite for the differences observed in miR-21 expression levels between the SiHa and HaCaT cells, and that AP-1 transcription factor is likely cooperating with other factors to achieve higher levels of expression of this promoter upon PMA treatment. These data suggest that this in vivo interaction contributes to miR-21 gene regulation during the cervical carcinogenesis process. Previous studies have noted that the oncogenic or tumor suppressive activity exhibited by distinct members of AP-1 transcription factor is dependent on cell context, genetic background of the tumor, and on AP-1 composition when it binds to non-canonical recognition sequences [26, 27]. Because of these properties, the AP-1 complex has been described as a double-edged sword in tumorigenesis [28, 29]. Furthermore, it has been reported that miR-21 promoter has recognition sites for other transcription factors such as STAT3 and NF-kB; which can regulate miR-21 gene expression at the transcriptional level during carcinogenesis [30, 31]. Our results confirm that c-Fos binds in vivo to AP1D, AP1M, and AP1P sequences in the miR-21 promoter and we propose that it is involved in the AP-1/miR-21 regulatory genetic network during cervical cancer development.

In this study, we demonstrated that the three AP-1 recognition sequences of the miR-21 promoter have promoter activity in cervical cancer cells, and we found that this activity decreased when the AP-1 sequences are deleted in a progressive manner, demonstrating a synergistic effect since the presence of AP-1 sequences increased miR-21 promoter activity (Fig. 7). The demonstration that AP-1 binding sites belong to the miR-21 signal transduction pathway [32], leads us to raise the hypothesis that these molecules are involved in induction of miR-21 gene expression in cervical cancer cells. The AP-1 transcription factor interacts constitutively with the miR-21 core promoter to generate a level of transcription dependent of external stimuli in the context of regulatory genetic networks [33]. These transcriptional complexes could associate with AP-1 recognition sequences and cooperate with other adjacent transcription factors, enhancing the stability of the transcriptional machinery, or alternatively recruiting co-activators that would increase miR-21 promoter activity throughout the autoregulatory feedback loop, as has been reported previously [32]. In addition, Ferguson et al. [34], demonstrated that the transcriptional activation of recombinant AP-1 in a synthetic promoter that has AP-1 recognition sites, can act on its own or interact with other proteins to increase transcription of the miR-21 promoter. In this context, it has been demonstrated that both c-Fos and c-Jun binding to the miR-21 promoter region increases after treatment with PMA, and that STAT3 and AKT signal transduction pathways can induce miR-21 gene promoter activity in cancer cells [35, 36]. We showed that AP-1 recognition sequences from the miR-21 core promoter functionally cooperate and this scenario was supported by the observation that deletion of three AP-1 binding sites decreased the transcription levels of miR-21 in the context of the AP-1/miR-21 regulatory genetic network. Our findings do not exclude that other molecular pathways mediated by different regulatory binding sites may also be present in the miR-21 core promoter region, and may contribute to its transcriptional regulation. Therefore, there exist excellent opportunities to identify new therapeutic molecular targets against cervical cancer.

Full understanding of the interplay between AP-1-dependent transcriptional control and miR-21-mediated post-transcriptional regulation will require study of the regulatory genetic network in physiological conditions and in development of pathology, as well as the identification of new targets for therapeutic intervention. In this context, several reports have contributed strong evidence in the analysis of gene regulation of the AP-1/miR-21 functional circuit. For instance, Fujita et al. [32], described that AP-1 activates miR-21 transcription in conjunction with SWI/SNF complex. They concluded that a conserved double-negative feedback regulatory mechanism might be occurring to sustain miR-21 gene expression in HL-60 cells. Talotta et al. [37], reported that miR-21 is induced by AP-1 in response to the Ras oncoprotein in rat thyroid cells. The authors provide evidence of a positive autoregulation mechanism of AP-1 in the context of Ras oncogene transformation, as well as of the function of miR-21 as an essential target and regulator of the AP-1 complex in tumorigenesis. Du et al. [38], reported that BMP-6 inhibited miR-21 promoter activity by repressing EF1 and AP-1, and that BMP-6 has a role as an anti-metastasis factor through a mechanism involving transcriptional repression of miR-21 in breast cancer cells. Misawa et al. [39], described the role of miR-21 in maintenance of the chemoresistant phenotype of cancer cells through the involvement of AP-1 and PDCD4. Zhu et al. [40], studied the role of miR-21 in hepatocellular carcinoma cells. They demonstrated that miR-21 promotes the migration and invasion processes in hepatocellular carcinoma cells through the miR-21/PDCD4/AP-1 feedback loop, which may represents a therapeutic target in this malignancy. Zhang et al. [41], examined miR-21 expression in human cirrhotic liver samples. They found that miR-21 expression is maintained itself at constant high levels by using the miR-21/PDCD4/AP-1 circuit reported also by Zhu [40]. These findings indicate that the miR-21/PDCD4/AP-1 regulatory genetic network is one of the main driving forces in progression of hepatic fibrosis. This functional circuit comprises the regulatory genetic network of miR-21 in a positive feedback loop through which down-regulation of PTEN and PCDC4 keeps STAT3 activity under control, as has been previously described [42, 43]. Moreover, it has also been shown that microRNA genes can be regulated by epigenetic mechanisms such as aberrant histone post-transcriptional modification or hypermethylation of CpG islands [44, 45]. Given the existing evidence, it is certainly conceivable that miR-21 gene expression might also be regulated by AP-1 transcription factor via methylated DNA sequence motifs. Our data reported herein in cervical cancer cell lines, and the evidence reported by other groups in tumor tissues, suggest that the regulatory genetic networks such as miR-21/PDCD4/AP-1 and miR-21/Let-7a/STAT3/PTEN, are highly complex and create a balance mechanism [35, 46]. In summary, the evidence described supports the existence of a universal AP-1/miR-21/target genes regulatory genetic network, which very likely also operates in cervical cancer cells. These regulatory genetic networks can be used as biomarkers in cervical cancer; however, it is important to be cautious because gene expression can be highly variable within cervical tissues in different subpopulations. Future studies could build on our results to identify more core factors, relevant motifs, and other parallel regulatory genetic networks in cervical carcinogenesis and/or other tumorigenesis processes.

## Conclusions

In conclusion, the oncogenic transformation process is generally associated with increased activity of endogenous transcription factors such as AP-1, which have a role in the regulation of expression of microRNAs such as miR-21 through several signal transduction pathways, and enhancement of miR-21 expression can contribute to oncogenic potential. This scenario supports the idea that microRNAs operate cooperatively with transcription factors in regulating a set of target genes, allowing coordinated regulation of gene expression, both at the transcriptional and post-transcriptional level. Therefore, our data strengthens the role of an interesting regulatory genetic network involving AP-1 and miR-21, which modulates critical genes in cervical cancer cells. Eventually, this knowledge may enable both AP-1 and miR-21 to be exploited as therapeutic targets in the treatment of cervical cancer.

## Data Availability

Not applicable.

## Abbreviations

3′-UTRs: 3′-untranslated regions
AP-1: activator protein 1
ATCC: American type culture collection
ChIP-PCR: chromatin immunoprecipitation-polymerase chain reaction
EMSA: electrophoretic mobility shift assay
HPV: human papillomavirus
miR-21: microRNA-21
PMA: phorbol-12-myristate-13-acetate
SR11302: (E,E,Z,E)-3-methyl-7-(4-methylphenyl)-9-(2,6,6-trimethyl-1-cyclohexen-1-yl)-2,4,6,8-nonatetraenoic acid
TFSEARCH: searching transcription factor binding sites
